# Adult height in relation to risk of cancer in a cohort of 22,809,722 Korean adults

**DOI:** 10.1038/s41416-018-0371-8

**Published:** 2019-02-19

**Authors:** Yoon Jin Choi, Dong Ho Lee, Kyung-Do Han, Hyuk Yoon, Cheol Min Shin, Young Soo Park, Nayoung Kim

**Affiliations:** 1 Department of Internal Medicine, Seoul National University Bundang Hospital, Seongnam, South Korea; 2 Department of Internal Medicine, Korea University Guro Hospital, Seoul, South Korea; 3 Department of Internal Medicine and Liver Research Institute, Seoul National University College of Medicine, Seoul, South Korea; 4 Department of Biostatistics, College of Medicine, The Catholic University of Korea, Seoul, South Korea

**Keywords:** Epidemiology, Risk factors

## Abstract

**Background:**

The present study examined whether adult height was associated with all site-combined or site-specific cancers.

**Methods:**

We used a nationwide claim data of 22,809,722 Korean participants including both men and women (2009–2012). The deciles of height from different age and sex groups were merged into a new quintile. We used Cox proportional hazards model to estimate hazard ratios (HRs) and 95% confidence intervals.

**Results:**

During a 5-year follow-up period, 765,651 patients were diagnosed with cancer. Height was positively associated with risk of all site-combined cancers and with malignancy in the oral cavity, larynx, lung, stomach, colorectum, liver, pancreas, biliary tract and gallbladder, breast, ovary, cervix and corpus uteri, prostate, testes, kidney, bladder, central nervous system, thyroid, skin, and lymphatic and haematopoietic systems. The HRs for all-site cancers per 5 cm increment in height was 1.09 and that of each site was the highest in thyroid, breast, lymphoma, testicular, and renal cancers. This association was more prominent in women and male non-smokers than in other counterparts.

**Conclusions:**

Taller adult height was significantly related to an increased risk of most cancers including neoplasm in the gallbladder or biliary tract and testes, but except for oesophagus.

## Introduction

Cancer was the second leading cause of death behind cardiovascular diseases. While the association between obesity (or visceral obesity) and cancer has been exhaustedly evaluated, the effect of height on the risk of having malignancy has received far less attention. Some early studies have reported that height is associated with an increased risk of all cancers and of specific cancers at various anatomic sites including the breast, prostate, and colorectum,^1–3^ the results were inconsistent according to gender, smoking status or body mass index (BMI).^4–7^ However, literature that included both sexes and a controlled possible compounder, such as weight or smoking, is limited, and most studies were conducted in Western countries. Based on this background, we aimed to investigate the association between attained adult stature and development of cancer using Korean nationwide databases. Additionally, whether different gender or smoking status could affect the association was evaluated.

## Methods

### Data source

Approximately 97% of the Korean population is registered with the National Health Insurance Corporation (NHIC), a single public health insurance programme, managed by the Korean government. NHIC subscribers undergo mandatory standardised medical examinations at least biennially from 40 years of age. Korean researchers can use the NHIC database with an approval by the official review committee.

From the total population of the Republic of Korea, we recruited 17,391,531 subjects who were older than 20 years and who underwent an initial baseline health check-up by the NHIS between 2009 and 2012. This cohort was then followed up until 2015. From this initial population, we excluded 1,108,167 subjects whose data was missed and 21,484,529 subjects who underwent more than two health check-ups during this period. Finally, 22,809, 722 subjects were included in the baseline cohort and followed up after excluding 448,040 who were diagnosed with any kind of cancer during the preceding years.

Medical record data and standardised self-reporting questionnaires were evaluated. Questionnaires at baseline included cancer risk factors; age (years), sex, smoking, drinking, physical activity and residency (rural and urban). Anthropic variables including blood pressure, height, weight, waist circumference, and values of serum fasting glucose and total cholesterol (mg/dL) were measured.

All procedures involving human participants were performed in accordance with the ethical standards of the institutional and national research committees and the 1964 Helsinki declaration including its later amendments or comparable ethical standards. This study was approved by the Institutional Review Board of Seoul National University Bundang Hospital (X-1608/360-904). Since the study involved routinely collected data, informed consent was not specifically obtained for this study.

### Study outcomes

The primary endpoint was newly diagnosed cancer at one of 23 different sites, which was defined using the International Classification of Diseases, 10th revision (ICD-10) codes: oral cavity and pharynx (ICD-10 C00–C14), larynx (C32), oesophagus (C15), stomach (C16), colorectum (C18–20), pancreas (C25), gallbladder and biliary tract (C23–24), lung (C33–34), breast (C50), corpus uteri (C54), cervix uteri (C53), ovary (C56), kidney (C64), bladder (C67), central nervous system (C70–72), skin (C43), non-Hodgkin lymphoma (NHL) (C82–86), multiple myeloma (C90), and leukaemia (C91–95). Codes for the reimbursement for serious diseases were also reviewed to reduce the error in studies with claim data; that is, both codes were required for the definition of cancer patients. The diagnosis was considered new when the patient had no such diagnosis before 2009. All patients with a previous malignancy were excluded. The same method (based on ICD and codes for the reimbursement for cancer) was applied to exclude persons who had history of any malignancy before index date.

### Definition of comorbidity and other variables

Regular exercise was defined as engaging in vigorous exercise on a regular basis (periods of high-intensity activity more than three times per week or periods of moderate intensity activity more than five times per week).^8^ BMI and systolic and diastolic blood pressure (mmHg) were also measured. Subjects were considered as obese when the BMI was ≥ 25 kg/m^2^ based on criteria for the Asian-Pacific region.^9^ Waist circumference ≥ 90 cm was defined as abdominal obesity.^10^ Diabetes mellitus (DM) was defined based on using insulin or oral hypoglycaemic agents, or a fasting plasma glucose level ≥ 126 mg/dL.^11^ Participants were diagnosed as being hypertensive if the systolic pressure was ≥ 140 mmHg, if the diastolic pressure was ≥ 90 mmHg, or if current antihypertensive medication was used. After overnight fasting for at least 8 h, blood specimens collected from each subject were processed and transported in cold storage to the Central Testing Institute (Neodin Medical Institute, Seoul, Korea). All the blood samples were analysed within 24 h after transportation.

Because height is dependent on age and sex, we divided the population into deciles of height for each age group (20–29 years, 30–39 years, 40–49 years, 50–59 years, 60–69 years and ≥70 years) and sex (Table S1).^12^ The deciles from the 12 different age and sex groups were merged into new quintiles of height. We analysed these merged quintile groups for incidence and hazard ratio of cancers.

### Statistical analyses

Data are presented as mean ± standard deviation for continuous variables and as proportions for categorical variables. Continuous variables were evaluated using analysis of variance (ANOVA), and categorical variables were evaluated using chi-square tests. A Cox proportional hazards model was used to determine the independent effect of height on all sites-combined and site-specific cancer risk, after controlling for age (continuous), sex, BMI, smoking status, alcohol consumption, physical activity, and diabetes. We compared the HR of the highest quintile of height compared with that of the lowest. Additionally, we evaluated the hazard ratios (HRs) in 5 cm increment in height. In Forrest plots, the HRs was represented by lines.

Statistical analyses were performed using SAS version 9.4 (SAS Institute, Cary, NC, United States) and R version 3.2.3 (The R Foundation for Statistical Computing, Vienna, Austria, http://www.Rproject.org). A two-sided *P*-value of <0.05 was considered to indicate statistical significance.

## Results

### Characteristics of the study population

A total of 22,809,722 participants among enrolees from 2009 through 2012 were finally analysed and followed up until 2015. They are grouped into the sex and age-adjusted quintile of height, and baseline characteristics at the enrolment were described in Table 1.

**Table 1:** Baseline characteristics by quintiles of height in study participants of the Korean National Health Insurance Corporation

	Height^a^				
Variables	Q1	Q2	Q3	Q4	Q5
	*N* = 4,676,674	*N* = 4,500,332	*N* = 4,529,559	*N* = 4,546,436	*N* = 4,556,721
Male sex (%)	2,370,686 (50.69)	2,272,941 (50.51)	2,233,313(49.31)	2,366,115 (52.04)	2,364,553 (51.89)
Rural place	2,683 895 (57.44)	2,469 988 (54.92)	2,437,470 (53.84)	2,405,326 (52.94)	2,347,919 (51.55)
Current smoking	1,130 426 (24.17)	1,113 803 (24.75)	1,092,698 (24.12)	1,172,094 (25.78)	1,161,493 (25.49)
Heavy drinking	272,158 (5.82)	290,868 (6.46)	290,388 (6.41)	328,429 (7.22)	344,478 (7.56)
Exercise (yes)	2,095,141 (44.8)	2,192,003 (48.71)	2,259,801(49.89)	2,353,225 (51.76)	2,408,444 (52.85)
BMI ≥ 25 kg/m^2^	1,532 624 (32.77)	1,460,203 (32.45)	1,437,396 (31.73)	1,451,344 (31.92)	1,429,447 (31.37)
Hypertension	1,280 811 (27.39)	1,178,929 (26.2)	1,179 257 (26.03)	1,152,861 (25.36)	1,176,424 (25.82)
Diabetes	437,274 (9.35)	408,901 (9.09)	411,955 (9.09)	407,450 (8.96)	430,391 (9.45)
Dyslipidemia	937,569 (20.05)	884,599 (19.66)	870,488 (19.22)	851,624 (18.73)	835,069 (18.33)
Age (year)^b^	48.1 ± 15.0	47.5 ± 14.2	47.7 ± 14.2	46.9 ± 14.0	47.2 ± 14.3
Age (median, IQR)	48 (37–58)	47 (38–57)	48 (37–57)	46 (37–56)	46 (36–58)
Height (cm)^b^	155.8 ± 7.7	160.6 ± 7.3	163.2 ± 7.3	166.6 ± 7.5	171.2 ± 8.0
Weight (kg)^b^	57.8 ± 9.9	61.4 ± 10.4	63.3 ± 10.8	65.9 ± 11.4	69.4 ± 12.4
BMI (kg/m^2^)^b^	23.8 ± 3.3	23. 8 ± 3.3	23.7 ± 3.2	23.7 ± 3.3	23.6 ± 3.3
Waist circumference (cm)^b^	78.3 ± 8.8	79.3 ± 9.0	79.9 ± 9.1	80.7 ± 9.3	82.0 ± 9.7
Glucose^b^	97.4 ± 23.7	97.4 ± 23.4	97.4 ± 23.2	97.5 ± 23.2	97.9 ± 23.5
Cholesterol^b^	196.2 ± 37.5	195.7 ± 37.1	194.9 ± 36.8	194.2 ± 36.6	192.9 ± 36.3
Diastolic BP^b^	76.1 ± 10.2	76.1 ± 10.2	76.0 ± 10.1	76.1 ± 10.0	76.1 ± 10.0
Systolic BP^b^	122.5 ± 15.7	122.2 ± 15.3	122.1 ± 15.2	122.1 ± 14.9	122.3 ± 14.8

*BMI* body mass index, *BP* blood pressure, *IQR* interquartile range, *Q* quintile

Values are presented as number (%)

^a^Newly combined quintile from stratified height groups dividing it by age (10 years old) and by gender in order to minimise the effect of nutritional changes over time on the height (see Supplementary table 1)

^b^Mean ± standard deviation

Approximately, half of the total enrolees were men. Waist circumference and weight increased when the height increased, while BMI deceased. The frequencies of hypertension and dyslipidaemia tended to decrease when the quintile of height increased (*P* < 0.001 for trend). Taller participants were likely to live in urban places, consume large amount of alcohol, smoke currently, and be more physically active than shorter persons.

### Cancer risk stratified by height

Taller adult height was associated with increased risks for total combined-cancers. Compared to participants in the lowest quintile, those in the highest quintile had a 28% increased risk after controlling age, sex, current smoking, current alcohol consumption, regular physical activity, DM and BMI (HR 1.28, 95% CI: 1.27–1.28, *P* for trend < 0.001) (Table 2).

**Table 2:** Incidences and adjusted hazard ratios of all-23 cancers and 10 representative site-specific cancers

Subtype	Height	Event	Duration	IR	HR (95% CI)
All-cancers	Q1	144,261	24,885,162	5.80	1 (reference)
	Q2	143,653	23,942,004	6.00	1.09 (1.08,1.10)
	Q3	157,825	23,965,194	6.59	1.18 (1.17,1.19)
	Q4	152,780	24,010,306	6.36	1.18 (1.18,1.19)
	Q5	167,132	23,857,126	7.01	1.28 (1.27,1.28)
Lung	Q1	14,037	25,237,573	0.56	1 (reference)
	Q2	13,229	24,301,233	0.54	1.10 (1.07,1.12)
	Q3	14,823	24,360,683	0.61	1.19 (1.16,1.22)
	Q4	13,914	24,396,653	0.57	1.21 (1.18,1.24)
	Q5	15,789	24,278,177	0.65	1.31 (1.28,1.34)
Breast	Q1	12,598	12,382,034	1.02	1 (reference)
	Q2	13,850	11,938,750	1.16	1.15 (1.12,1.18)
	Q3	15,979	12,235,058	1.31	1.30 (1.27,1.33)
	Q4	15,513	11,591,065	1.34	1.34 (1.31,1.37)
	Q5	16,640	11,555,955	1.44	1.45 (1.42,1.49)
Colorectum	Q1	28,415	25,187,463	1.13	1 (reference)
	Q2	27,905	24,251,652	1.15	1.09 (1.08,1.11)
	Q3	30,385	24,307,787	1.25	1.16 (1.15,1.18)
	Q4	29,414	24,344,214	1.21	1.18 (1.17,1.20)
	Q5	32,348	24,220,381	1.34	1.26 (1.24,1.28)
Prostate	Q1	9985	12,819,866	0.78	1 (reference)
	Q2	9532	12,323,481	0.77	1.11 (1.08,1.15)
	Q3	11,059	12,080,481	0.92	1.20 (1.17,1.23)
	Q4	9860	12,762,932	0.77	1.18 (1.15,1.22)
	Q5	11,787	12,675,330	0.93	1.30 (1.26,1.33)
Stomach	Q1	27,067	25,184,646	1.07	1 (reference)
	Q2	25,201	24,252,220	1.04	1.04 (1.03,1.06)
	Q3	26,790	24,309,880	1.10	1.08 (1.061,1.10)
	Q4	25,362	24,348,674	1.04	1.08 (1.06,1.10)
	Q5	27,262	24,228,267	1.13	1.11 (1.09,1.13)
Liver	Q1	12,796	25,236,793	0.51	1 (reference)
	Q2	11,790	24,301,084	0.49	1.02 (1.00,1.05)
	Q3	12,253	24,363,395	0.50	1.03 (1.01,1.06)
	Q4	11,719	24,398,463	0.48	1.04 (1.01,1.06)
	Q5	12,572	24,281,779	0.52	1.07 (1.04,1.09)
Cervix uteri	Q1	3241	12,408,168	0.26	1 (reference)
	Q2	3105	11,969,000	0.26	1.02 (0.97,1.07)
	Q3	3425	12,270,201	0.28	1.10 (1.04,1.15)
	Q4	3241	11,625,391	0.28	1.10 (1.05,1.16)
	Q5	3178	11,593,623	0.27	1.09 (1.04,1.15)
Oesophagus	Q1	1859	25,257,664	0.07	1 (reference)
	Q2	1643	24,321,065	0.07	1.03 (0.97,1.10)
	Q3	1825	24,383,568	0.07	1.09 (1.02,1.17)
	Q4	1587	24,418,442	0.06	1.03 (0.96,1.10)
	Q5	1779	24,303,222	0.07	1.08 (1.01,1.15)
Bladder	Q1	3794	25,252,217	0.15	1 (reference)
	Q2	3737	24,315,189	0.15	1.14 (1.09,1.19)
	Q3	4190	24,377,074	0.17	1.27 (1.17,1.27)
	Q4	4013	24,411,826	0.16	1.27 (1.21,1.33)
	Q5	4420	24,296,165	0.18	1.31 (1.26,1.37)
Lymphoma	Q1	2627	25,255,323	0.10	1 (reference)
	Q2	2676	24,317,982	0.11	1.11(1.05,1.17)
	Q3	3092	24,380,017	0.13	1.26 (1.20,1.33)
	Q4	3151	24,414,253	0.13	1.33 (1.26,1.40)
	Q5	3708	24,298,211	0.15	1.53 (1.46,1.61)

*CNS* central nervous system, *IR* incidence rate, *Q* quintiles, *HR* Hazard ratio, *CI* confidence interval

^a^Newly combined quintile from stratified height groups dividing it by age (10 years old) and by gender

^b^Age, sex, body mass index, current smoking, current alcohol consumption, regular physical activity and diabetes mellitus

**Fig. 1:**
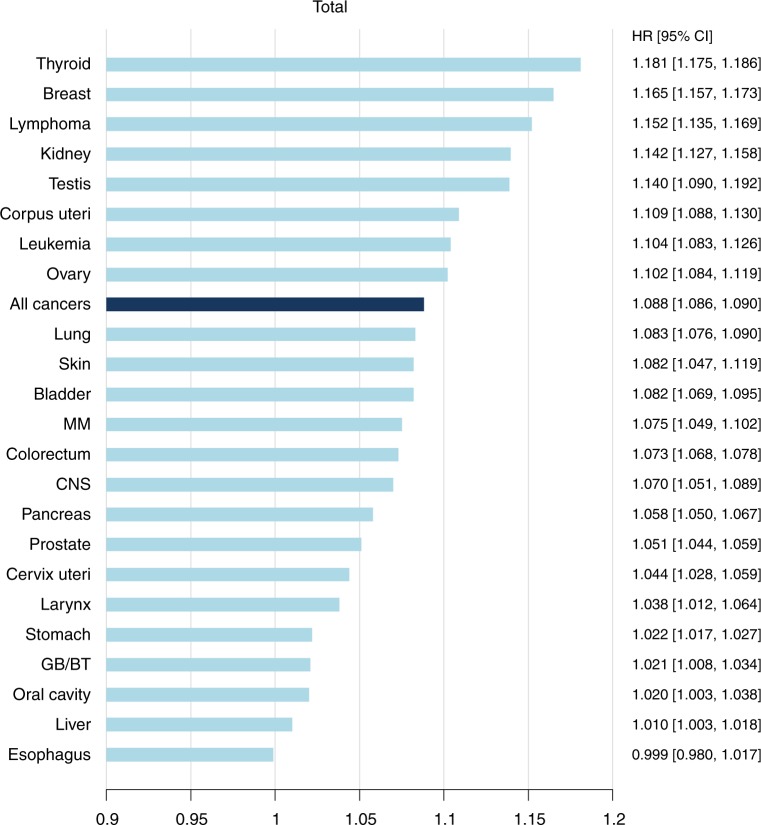
Hazard ratios and 95% confidence intervals per 5 cm increase in height for all cancers and 23 site-specific cancers. HRs are adjusted for age, sex, body mass index, current smoking, current alcohol consumption, regular physical activity, and presence or absence of diabetes mellitus. *HR* hazard ratio, *CI* confidence interval, *MM* multiple myeloma, *CNS* central nerve system, *GB* gallbladder, *BT* biliary tract

The HR of all-sites cancers per 5 cm increase in height was 1.09 (95% CI 1.086–1.090) (Fig. 1). Taller adult stature was positively associated with increased risks of cancers at all 23 anatomic sites in a multivariable model that included BMI (Table 2 and Table S2). Compared to persons in the shortest group, HRs and 95% CIs of persons in the tallest group from the multivariable model were as follows: oral cavity (1.09, 95% CI: 1.03–1.15), larynx (1.19, 95% CI: 1.09–1.30), stomach (1.11, 95% CI: 1.09–1.13), colorectum (1.26, 95% CI: 1.24–1.28), lung (1.31, 95% CI: 1.28–1.34), pancreas (1.22, 95% CI: 1.18–1.25), thyroid (1.57, 95% CI: 1.54–1.59), kidney (1.56, 95% CI: 1.48–1.63), bladder (1.31, 95% CI: 1.26–1.37), multiple myeloma (1.29, 95% CI: 1.18–1.40), lymphoma (1.42, 95% CI: 1.35–1.50), leukaemia (1.34, 95% CI: 1.26–1.44), CNS (1.24, 95% CI: 1.16–1.31), melanoma (1.30, 95% CI: 1.17–1.46), breast (1.45, 95% CI: 1.42–1.49), ovary (1.27, 95% CI: 1.21–1.34), prostate (1.30, 95% CI: 1.26–1.33) and testes (1.62, 95% CI: 1.38–1.91). Particularly, there was a dose-dependent increase in the risk of cancers in oral cavity, liver, colorectum, lung, pancreas, breast, ovary, thyroid, bladder, kidney, melanoma, and haematopoietic system (Table 2 and Table S2). With regard to oesophageal cancer, the tallest group had an increased risk for the disease compared with the shortest group; there was a no dose-dependent relationship. HRs for cancers per 5 cm increase in height were highest in thyroid, breast, lymphoma, testicular, and renal cancers (Fig. 1), while oesophageal cancer did not show a significant association due to a lack of a dose-dependent association.

### Combined effect of height on the development of cancers according to gender and current smoking status

The baseline characteristics were presented by gender (Table S3). Men were more likely to smoke, drink excessively, exercise regularly, and have obesity, hypertension, and diabetes compared to women, while women were older than males with the same height rank, more women lived in cities, and had hyperlipidaemia.

**Fig. 2:**
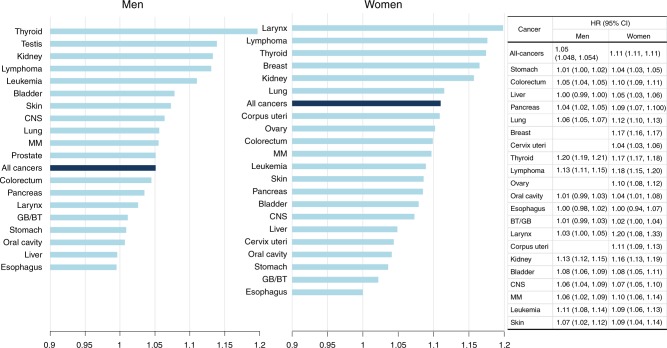
Hazard ratios per 5 cm increase in height for all cancers and cancers at 23 different sites, stratified by gender. HRs are adjusted for age, body mass index, current smoking, current alcohol consumption, regular physical activity, and presence or absence of diabetes mellitus. *HR* hazard ratio, *CI* confidence interval, *MM* multiple myeloma, *CNS* central nerve system, *GB* gallbladder, *BT* biliary tract

HR of combined all-sites cancers was more prominent in women than in men (women: HR 1.11 95% CI: 1.107–1.114 versus men: HR 1.05, 95% CI: 1.048–1.054) (Fig. 2) Among men, the magnitude of association was highest in thyroid cancer (HR 1.20, 95% CI: 1.19–1.21), while cancers in the larynx and lymphatic system were most associated with height in women (1.20, 95% CI: 1.08–1.33; 1.18, 95% CI: 1.15–1.20) (Fig. 2).

**Fig. 3:**
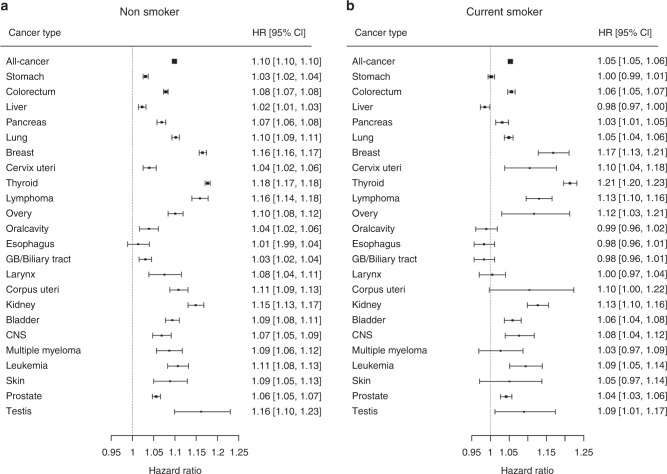
Hazard ratios and 95% confidence intervals per 5 cm increase in height for all cancers and cancers at 23 different sites, stratified by smoking status at enrolment. HRs are adjusted for age, sex, body mass index, current alcohol consumption, regular physical activity, and presence or absence of diabetes mellitus among (**a**) non-smoker and (**b**) current-smoker. *HR* hazard ratio, *CI* confidence interval, *CNS* central nerve system, *GB* gallbladder

The effect of smoking status on the development of all cancers and site-specific cancers was analysed (Fig. 3). Current smoking reduced the magnitude of the association between taller height and the development of all combined-cancer by ~5% compared to non-smoking (non-smoker: 1.10 vs. current smoker: 1.05). Among non-smokers, there was a significant association between increased risk for oral cavity cancer and taller stature. The magnitudes of the association between stature and risk for cancers in oral cavity, larynx, lung, corpus and cervix uteri, stomach, liver, pancreas, multiple myeloma, and melanoma were decreased among current smokers compared with non-smokers (Fig. 3).

When each gender was stratified by smoking status, the magnitudes of the association in female were generally larger than those in male irrespective of smoking status (Table S4).

Among men, non-smoker showed a stronger association between taller height and most cancers compared to smokers, but this trend was weak among women (Table S4).

## Discussion

In the present nationwide study, which involved a large cohort of Korean individuals, we found that height was positively associated with risk of all sites-combined cancer and with cancers of the larynx, stomach, colorectum, pancreas, biliary tract and gallbladder, lung, liver, breast, ovary, cervix and corpus uteri, kidney, bladder, prostate, testes, CNS, thyroid, skin (excluding non-melanoma), and lymphatic and haematopoietic systems. The HR for all-site cancers per 5 cm increment in height was 1.09 (95% CI: 1.086–1.090), and the magnitude of the associations for specific sites ranged from HR 1.01 (95% CI: 1.00–1.02) for liver cancer to HR 1.18 (95% CI: 1.18–1.19) for thyroid cancer. This association was more prominent in women and non-smokers than in other counterparts.

Several studies have evaluated the association between attained stature and the risk for development of cancer,^2–7^ showed generalised outcomes, i.e., cancer risk increased with increase in stature. For instance, the Million Women Study,^7^ which is one of the largest studies, presented the positive associations between height and cancers in the colon, rectum, breast, endometrium, ovary, kidney, CNS, skin, and lymphatic and haematopoietic systems. In accordance with the result, our study showed significant associations for all above-mentioned sites. Furthermore, we newly reported positive relationships between taller stature and increased vulnerability to thyroid, gallbladder and biliary tract, and testicular cancers, which the Million Women Study did not report. A previous Korean study has also reported the association of greater stature with a higher risk of biliary cancers in line with our results.^5^ A recent Danish study has reported that the risk for testicular germ cell tumour was associated with height by age 7 years, not between ages 7 and 13.^13^ By contrast, the present study showed that taller adult height was significantly associated with a higher chance of testicular cancers in adults. A meta-analysis on the association between adult height and malignancy in testes showed a positive result.^14^

One of the possible mechanisms which link height to cancers is that the adult height correlates with organ sizes.^15^ According to this hypothesis, more active cell proliferation for the organs in taller persons could increase the possibility of mutation. Another explanation is an increased level of insulin-like growth factor (IGF) which correlates with calorie intake in animals,^16^ height in children,^17^ and risk of colorectal and prostate cancer in adult humans.^18^ In support of the IGF-malignancy link, there is a positive relationship of stature with carcinoma of the prostate and colorectum in several studies. Some genetic factors linked with height might share mechanisms underlying tumour vulnerability. Recently, Lophatananon et al.^19^ has reported that height and several genetic variants related to the human growth pathway, such as IGF or growth hormone secretagogue receptor,^20^ are associated with high-grade prostate cancer risk.

Although gastric, hepatic and cervical cancers traditionally thought to be related with infection, there have been some reports that obesity or metabolic syndrome are associated with the increased risk of them.^21–25^ For instance, hepatic cellular carcinoma can develop in noncirrhotic livers with non-alcoholic fatty liver disease, particularly in the presence of multiple metabolic risk factors, such as obesity and diabetes.^23^ Obese women might receive screening for cervical cancer less frequently than normal-weight women.^25^ Therefore, it is not possible to exclude the possibility that these tumours are linked to IGF hypothesis.

According to literature, the association of height with all-sites cancers was statistically significant after adjustment for potential confounding factors except for the current smoking and usage of hormone therapy.^6,26^ Our results confirmed that current smoking history is an important effect modifier; that is, height was more strongly related to cancer risk among non-smokers than among smokers.^7,27^ With regard to gender, similar associations in both genders have been reported.^26^ Slightly prominent associations among women than among men were shown in the present study. Given that this trend is maintained regardless of whether or not smoking, gender as well as smoking can be a modifier affecting the association between development of height and cancer. To date, no specific mechanism has been known as to whether the association between tall stature and the development of cancer is greater in women or non-smokers.

Another possible modifier is BMI. Weight is correlated with height and could confound the association between height and cancer.^28,29^ As for oesophageal cancer, which is negatively associated with BMI, the association with height was not significant.

There is some degree of consistency in the impact size of stature on site-specific cancer risks. For instance, melanoma, leukaemia, kidney cancer, and colorectal cancer have been ranked in the highest positions among both sexes.^5–7,27^ When the gender was restricted to men, the Whitehall cohort^4^ showed melanoma and kidney cancers were the most height-associated, while Sung et al.^5^ reported thyroid cancer, lymphoma, and melanoma as such. While only our study and the study by Sung et al.^5^ showed the highest association with taller stature in thyroid cancer, in the present study, skin cancers did not represent the strong association. Ethnic differences may have affected this result because thyroid cancer has been South Korea’s most common cancer, but skin cancers rarely occur in Korea.^30^ Additionally, a significant association with cancers in the biliary tract and taller persons has been reported in the above two studies. Even though the two studies were based in Korea, there were some differences in results; in the present study, persons with taller stature were at a higher risk of developing stomach and pancreas cancers, while in the study by Sung et al. there was no such clear association between height and stomach and pancreatic cancer.^5^ This could be because the two studies included in populations from different time periods; thus, resulting in a younger generation in our study. *Heliobacter pylori*, one of the most potent factors in the carcinogenesis of stomach cancer, is declining over time, from 66.9% in 1998 to 54.4% in 2011^17^; Because *H. pylori* prevalence is decreasing in Korea and Japan, height may have a greater association with gastric cancer in younger individuals. On the other hand, malignancy in the pancreas has continuously increased, but the incidence is relatively low.^30^ A huge sample size of the present study could contribute the positive association unlike the study by Sung et al.^5^ Additionally, Sung et al. previously have shown a marked reduction in the positive association between height and liver cancer after an additional adjustment for hepatitis B viral antigenicity. Therefore, the significant association with liver cancer in taller persons in the present study should be interpreted cautiously because we did not adjust for hepatitis B or C viral antigenicity. Our results suggest a special caution when assessing the association of height with some cancers that are closely associated with weight or infection.

To determine the casualty between anthropometric traits and cancer, Mendelian randomisation study has been used.^31^ The basic concept is genetic factors influencing height play roles in carcinogenesis. One British study recently have reported that genetically determined taller height is causally associated with greater overall cancer susceptibility and cancer mortality by age 60.^32^ Further study is necessary to investigate which factors make the association or casualty greater among women or non-smoker.

One of the strengths of our present study is that we used the nationwide database for both sexes to perform a population-based cohort study. Some analyses involved small numbers of events for certain cancer sub-types; this reduced researchers’ ability to detect existing associations. To our knowledge, this is the first study that reported the positive relationship between adult height and cancers in testes among Asian population. This study evaluated as many as 23 site-specific cancers including and biliary tract and gallbladder.

The limitation of the present study is a short follow-up period and the lack of data about menstruation, parity, breast-feeding or use of exogenous hormones. Nonetheless, the result in this study is in accordance with previous studies with 10 years or more follow-up period^7^ and the consistent impact of height on the development of female organ cancers has been reported after controlling for reproductive factors.^26^ Authors did not exclude women who had undergone hysterectomy or bilateral oophorectomy at recruitment for the analyses of uterine and ovarian cancer.

Because the study participants were recruited based on health check-up examinee, it cannot be free from the potential selection bias. Further research on the exact mechanisms and potential lifestyle factors which may have a combined synergistic effect for the development of malignancies among individuals of taller stature could provide a clue for better cancer prevention.

## Supplementary information

Supplementary table 1

Supplementary table 2

Supplementary table 3

Supplementary table 4

## Data Availability

Data are available from the Korean National Health Insurance Corporation (https://nhiss.nhis.or.kr/bd/ab/bdaba002cv.do) for researchers who meet the criteria for access to confidential data. These are third party data. We did not have any special access privileges that others would not have. If the proposal is accepted by an evaluation committee of NHIC, the researcher would receive the de-identified NHIC dataset after paying some fee.
