# Supplementary material for: Adult height in relation to risk of cancer in a cohort of 22,809,722 Korean adults
Source: Br J Cancer. 2019 Feb 19;120(6):668–74. doi: 10.1038/s41416-018-0371-8 (PMC6462046; doi:10.1038/s41416-018-0371-8)
Supplement: Supplementary file 1 — Supplementary table 1 [file 41416_2018_371_MOESM1_ESM.docx]

Supplementary Table 1. Reference value of height deciles based on age and sex groups

| Sex | Age group  (year) | Number | Height (cm) | | | | | | | | | |
| --- | --- | --- | --- | --- | --- | --- | --- | --- | --- | --- | --- | --- |
|  |  |  | D1 | D2 | D3 | D4 | D5 | D6 | D7 | D8 | D9 | D10 |
| Men | 20-29 | 1,482,865 | <166 | 166-169 | 169-171 | 171-173 | 173-174 | 174-176 | 176-177 | 177-179 | 179-182 | >182 |
|  | 30–39 | 2,550,100 | <165 | 165-168 | 168-170 | 170-171 | 171-173 | 173-174 | 174-176 | 176-178 | 178-180 | >180 |
|  | 40-49 | 2,931,633 | <163 | 163-166 | 166-168 | 168-169 | 169-171 | 171-172 | 172-173 | 173-175 | 175-178 | >178 |
|  | 50-59 | 2,415,634 | <161 | 161-163 | 163-165 | 165-167 | 167-168 | 168-170 | 170-171 | 171-173 | 173-175 | >175 |
|  | 60-69 | 1,443,957 | <159 | 159-161 | 161-163 | 163-165 | 165-166 | 166-167 | 167-169 | 169-171 | 171-173 | >173 |
|  | ≥ 70 | 783,419 | <157 | 157-159 | 159-161 | 161-163 | 163-164 | 164-165 | 165-167 | 167-169 | 169-171 | >171 |
| Women | 20-29 | 1,376,677 | <155 | 155-157 | 157-159 | 159-160 | 160-161 | 161-163 | 163-164 | 164-166 | 166-168 | >168 |
|  | 30–39 | 1,313,309 | <153 | 153-156 | 156-157 | 157-159 | 159-160 | 160-161 | 161-163 | 163-165 | 165-167 | >167 |
|  | 40-49 | 3,141,983 | <151 | 151-154 | 154-155 | 155-157 | 157-158 | 158-159 | 159-161 | 161-162 | 162-165 | >165 |
|  | 50-59 | 2,637,473 | <149 | 149-151 | 151-153 | 153-154 | 154-156 | 156-157 | 157-158 | 158-160 | 160-162 | >162 |
|  | 60-69 | 1,635,966 | <147 | 147-149 | 149-151 | 151-152 | 152-153 | 153-155 | 155-156 | 156-158 | 158-160 | >160 |
|  | ≥ 70 | 1,096,706 | <142 | 142-145 | 145-147 | 147-148 | 148-150 | 150-151 | 151-153 | 153-154 | 154-157 | >157 |

D, decile
