# Supplementary material for: Adult height in relation to risk of cancer in a cohort of 22,809,722 Korean adults
Source: Br J Cancer. 2019 Feb 19;120(6):668–74. doi: 10.1038/s41416-018-0371-8 (PMC6462046; doi:10.1038/s41416-018-0371-8)
Supplement: Supplementary file 2 — Supplementary table 2 [file 41416_2018_371_MOESM2_ESM.docx]

Supplementary table 2. Baseline characteristics according to quintiles of height by gender in study participants

|  | Men | | | | |  | Women | | | | |
| --- | --- | --- | --- | --- | --- | --- | --- | --- | --- | --- | --- |
| Variables | Q1 | Q2 | Q3 | Q4 | Q5 |  | Q1 | Q2 | Q3 | Q4 | Q5 |
|  | n=2370686 | n=2272941 | n=2233313 | n=2366115 | n=2364553 |  | n=2305988 | n=2227391 | n=2296246 | n=2180321 | n=2192168 |
| Urban place | 991285 (41.8) | 1003507 (44.2) | 1010963 (45.3) | 1089060 (46.0) | 1118562 (47.3) |  | 997561 (43.3) | 1023869 (46.0) | 1078434 (47.0) | 1049534 (48.2) | 1087727 (49.7) |
| Current smoking | 1048026 (44.2) | 1027509 (45.2) | 998718 (44.7) | 1077452 (45.5) | 1057861 (44.7) |  | 82400 (3.6) | 86294 (3.9) | 93980 (4.0) | 94642 (4.3) | 103632 (4.7) |
| Heavy drinking | 252435 (10.7) | 269402 (11.85) | 268022 (12.0) | 305030 (12.9) | 318309 (13.5) |  | 19723 (0.9) | 21466 (0.96) | 22366 (1.0) | 23399 (1.1) | 26169 (1.2) |
| Exercise (yes) | 1198061 (50.5) | 1251256 (55.1) | 1257906 (56.3) | 1373654 (58.1) | 1395644 (59.0) |  | 897080 (39.0) | 940747 (42.2) | 1001895 (43.6) | 979571 (44.9) | 1012800 (46.2) |
| BMI≥25Kg/m^2^ | 840221 (35.4) | 844915 (37.2) | 833375 (37.3) | 898633 (38.0) | 915053 (38.7) |  | 692403 (30.0) | 615288 (27.6) | 604021 (26.3) | 552711 (25.4) | 514394 (23.5) |
| Hypertension | 658698 (27.8) | 625218 (27.5) | 631686 (28.3) | 640659 (27.1) | 672104 (28.4) |  | 622113 (27.0) | 553711 (24.9) | 547571 (23.9) | 512202 (23.5) | 504320 (23.0) |
| Diabetes | 249562 (10.5) | 236812 (10.4) | 240434 (10.8) | 242778 (10.3) | 260467 (11.0) |  | 187712 (8.1) | 172089 (7.7) | 171521 (7.5) | 164672 (7.6) | 169924 (7.8) |
| Dyslipidemia | 432179 (18.2) | 415736 (18.3) | 400943 (18.0) | 412856 (17.5) | 409894 (17.3) |  | 505390 (21.9) | 468863 (21.1) | 469545 (20.5) | 438768 (20.1) | 425175 (19.4) |
| Age (y)^a^ | 46.48 ± 14.43 | 46.25 ± 13.54 | 46.53 ± 14.44 | 45.61 ± 13.56 | 46.43 ± 13.88 |  | 49.77 ± 15.41 | 48.79 ± 14.65 | 48.93 ± 13.82 | 48.34 ± 14.41 | 48.03 ± 14.75 |
| Median age | 46 (36-56) | 45 (36-55) | 46 (34-57) | 44 (35-54) | 45 (35-56) |  | 49 (40-60) | 48 (40-58) | 48 (40-57) | 48 (40-58) | 48 (38-60) |
| Height (cm) ^a^ | 162.08 ± 4.2 | 167.1 ± 3.09 | 169.9 ± 3.26 | 173.05 ± 3.19 | 177.81 ± 4.2 |  | 149.29 ± 4.33 | 154.01 ± 3.37 | 156.73 ± 3.21 | 159.58 ± 3.32 | 164.06 ± 4.15 |
| Weight (Kg) ^a^ | 63.14 ± 8.87 | 67.54 ± 8.96 | 69.84 ± 9.36 | 72.61 ± 9.68 | 76.82 ± 10.64 |  | 52.38 ± 7.69 | 55.2 ± 7.7 | 56.94 ± 7.9 | 58.57 ± 8.12 | 61.42 ± 8.72 |
| BMI (Kg/m^2^) ^a^ | 24.01 ± 3.1 | 24.18 ± 3.05 | 24.18 ± 3.06 | 24.24 ± 3.08 | 24.28 ± 3.1 |  | 23.53 ± 3.48 | 23.3 ± 3.4 | 23.21 ± 3.34 | 23.03 ± 3.35 | 22.85 ± 3.35 |
| Waist circumference (cm) ^a^ | 81.14 ± 7.65 | 82.79 ± 7.58 | 83.67 ± 7.71 | 84.55 ± 7.79 | 86.26 ± 8.02 |  | 75.44 ± 8.99 | 75.81 ± 8.95 | 76.24 ± 8.88 | 76.54 ± 9.03 | 77.3 ± 9.21 |
| Glucose ^a^ | 99.26 ± 25.59 | 99.53 ± 25.43 | 99.54 ± 25.24 | 99.49 ± 25.1 | 100.08 ± 25.53 |  | 95.48 ± 21.45 | 95.24 ± 20.91 | 95.32 ± 20.71 | 95.25 ± 20.6 | 95.46 ± 20.91 |
| Cholesterol ^a^ | 194.69 ± 36.96 | 194.93 ± 36.5 | 193.61 ± 36.22 | 193.59 ± 36.07 | 192.57 ± 35.75 |  | 197.68 ± 38.04 | 196.41 ± 37.61 | 196.13 ± 37.27 | 194.79 ± 37.13 | 193.22 ± 36.79 |
| Diastolic BP ^a^ | 77.73 ± 9.9 | 77.91 ± 9.81 | 77.91 ± 9.7 | 78.01 ± 9.68 | 78.19 ± 9.63 |  | 74.48 ± 10.31 | 74.17 ± 10.16 | 74.1 ± 10.09 | 73.98 ± 10.01 | 73.91 ± 9.9 |
| Systolic BP ^a^ | 124.52 ± 14.67 | 124.55 ± 14.28 | 124.78 ± 14.16 | 124.64 ± 13.91 | 125.08 ± 13.81 |  | 120.37 ± 16.4 | 119.71 ± 15.92 | 119.51 ± 15.65 | 119.34 ± 15.5 | 119.26 ± 15.27 |

BMI, body mass index; BP blood pressure; Q, quintile

Values are presented as number (%)

^a^ mean ± standard deviation
