# Supplementary material for: Adult height in relation to risk of cancer in a cohort of 22,809,722 Korean adults
Source: Br J Cancer. 2019 Feb 19;120(6):668–74. doi: 10.1038/s41416-018-0371-8 (PMC6462046; doi:10.1038/s41416-018-0371-8)
Supplement: Supplementary file 3 — Supplementary table 3 [file 41416_2018_371_MOESM3_ESM.docx]

Supplementary table 3. Incidences and adjusted hazard ratios of 13 site-specific cancers (other than those in Table 2)

| Subtype | Height | Event | Duration | IR | HR (95% CI)^a^ |
| --- | --- | --- | --- | --- | --- |
| Pancreas | Q1 | 8914 | 25244764 | 0.35 | 1 (reference ) |
|  | Q2 | 8584 | 24308168 | 0.35 | 1.08 (1.045,1.109) |
|  | Q3 | 9349 | 24369658 | 0.38 | 1.14 (1.111,1.177) |
|  | Q4 | 8980 | 24404687 | 0.37 | 1.16 (1.126,1.194) |
|  | Q5 | 9776 | 24288318 | 0.40 | 1.22 (1.183,1.254) |
| Ovary | Q1 | 2631 | 12411435 | 0.21 | 1 (reference ) |
|  | Q2 | 2701 | 11971642 | 0.23 | 1.09 (1.033,1.15) |
|  | Q3 | 3034 | 12273192 | 0.25 | 1.20 (1.139,1.265) |
|  | Q4 | 2882 | 11627895 | 0.25 | 1.22 (1.154,1.283) |
|  | Q5 | 2972 | 11595745 | 0.26 | 1.27 (1.205,1.339) |
| Oral cavity | Q1 | 2230 | 25256552 | 0.09 | 1 (reference ) |
|  | Q2 | 2108 | 24319510 | 0.09 | 1.04 (0.98,1.104) |
|  | Q3 | 2195 | 24382251 | 0.09 | 1.06 (1,1.126) |
|  | Q4 | 2146 | 24416855 | 0.09 | 1.08 (1.013,1.141) |
|  | Q5 | 2241 | 24301813 | 0.09 | 1.09 (1.025,1.153) |
| BT & GB | Q1 | 4297 | 25254376 | 0.17 | 1 (reference ) |
|  | Q2 | 3758 | 24317981 | 0.15 | 1.01 (0.968,1.057) |
|  | Q3 | 4136 | 24380308 | 0.17 | 1.08 (1.036,1.128) |
|  | Q4 | 3723 | 24415178 | 0.15 | 1.05 (1.005,1.098) |
|  | Q5 | 4064 | 24299788 | 0.17 | 1.10 (1.055,1.149) |
| Thyroid | Q1 | 24955 | 25183262 | 0.99 | 1 (reference ) |
|  | Q2 | 28936 | 24232771 | 1.19 | 1.21(1.189,1.23) |
|  | Q3 | 32538 | 24284030 | 1.34 | 1.34 (1.32,1.364) |
|  | Q4 | 33020 | 24317247 | 1.36 | 1.41 (1.389,1.436) |
|  | Q5 | 36263 | 24192373 | 1.50 | 1.57 (1.54,1.59) |
| Larynx | Q1 | 986 | 25259176 | 0.04 | 1 (reference ) |
|  | Q2 | 871 | 24322167 | 0.04 | 1.02 (0.93,1.116) |
|  | Q3 | 1053 | 24384725 | 0.04 | 1.18 (1.079,1.284) |
|  | Q4 | 931 | 24419595 | 0.04 | 1.12 (1.027,1.23) |
|  | Q5 | 1049 | 24304366 | 0.04 | 1.19 (1.091,1.299) |
| Corpus uteri | Q1 | 1863 | 12412771 | 0.15 | 1 (reference ) |
|  | Q2 | 1990 | 11972894 | 0.17 | 1.14 (1.073,1.218) |
|  | Q3 | 2108 | 12274660 | 0.17 | 1.20 (1.124,1.273) |
|  | Q4 | 1904 | 11629896 | 0.16 | 1.16 (1.088,1.237) |
|  | Q5 | 2083 | 11597480 | 0.18 | 1.30 (1.218,1.381) |
| kidney | Q1 | 2818 | 25254330 | 0.11 | 1 (reference ) |
|  | Q2 | 3104 | 24316436 | 0.13 | 1.19 (1.131,1.253) |
|  | Q3 | 3463 | 24378353 | 0.14 | 1.31 (1.245,1.375) |
|  | Q4 | 3562 | 24412561 | 0.15 | 1.38 (1.315,1.452) |
|  | Q5 | 4114 | 24296393 | 0.17 | 1.56 (1.482,1.631) |
| CNS | Q1 | 2040 | 25257335 | 0.08 | 1 (reference ) |
|  | Q2 | 1964 | 24320375 | 0.08 | 1.04 (0.981,1.111) |
|  | Q3 | 2138 | 24382969 | 0.09 | 1.13 (1.059,1.195) |
|  | Q4 | 2134 | 24417338 | 0.09 | 1.16 (1.094,1.236) |
|  | Q5 | 2288 | 24302309 | 0.09 | 1.24 (1.163,1.311) |
| MM | Q1 | 1009 | 25259567 | 0.04 | 1 (reference ) |
|  | Q2 | 963 | 24322553 | 0.04 | 1.08 (0.991,1.183) |
|  | Q3 | 1043 | 24385252 | 0.04 | 1.15 (1.05,1.248) |
|  | Q4 | 1027 | 24419759 | 0.04 | 1.20 (1.101,1.311) |
|  | Q5 | 1136 | 24304812 | 0.05 | 1.29 (1.183,1.402) |
| Leukemia | Q1 | 1567 | 25258411 | 0.06 | 1 (reference ) |
|  | Q2 | 1526 | 24321263 | 0.06 | 1.05 (0.975,1.123) |
|  | Q3 | 1675 | 24383962 | 0.07 | 1.14 (1.061,1.218) |
|  | Q4 | 1749 | 24418259 | 0.07 | 1.22 (1.136,1.303) |
|  | Q5 | 1955 | 24303193 | 0.08 | 1.34 (1.256,1.435) |
| Skin | Q1 | 581 | 25260232 | 0.02 | 1 (reference ) |
|  | Q2 | 548 | 24323185 | 0.02 | 1.06 (0.942,1.189) |
|  | Q3 | 570 | 24385977 | 0.02 | 1.08 (0.963,1.214) |
|  | Q4 | 587 | 24420494 | 0.02 | 1.18 (1.05,1.322) |
|  | Q5 | 664 | 24305508 | 0.03 | 1.30 (1.165,1.457) |
| Testis | Q1 | 243 | 12843474 | 0.02 | 1 (reference ) |
|  | Q2 | 276 | 12345834 | 0.02 | 1.20 (1.013,1.431) |
|  | Q3 | 307 | 12106498 | 0.03 | 1.34 (1.131,1.584) |
|  | Q4 | 289 | 12786526 | 0.02 | 1.25 (1.051,1.479) |
|  | Q5 | 384 | 12703562 | 0.03 | 1.62 (1.383,1.908) |

CNS, central nervous system; BT, biliary tract; GB, gallbladder; MM, multiple myeloma; IR, incidence rate; Q, quintiles; HR, Hazard ratio; CI, confidence interval

^a^ Age, sex, **body mass index**, current smoking, current alcohol consumption, regular physical activity, and diabetes
