# Supplementary material for: Adult height in relation to risk of cancer in a cohort of 22,809,722 Korean adults
Source: Br J Cancer. 2019 Feb 19;120(6):668–74. doi: 10.1038/s41416-018-0371-8 (PMC6462046; doi:10.1038/s41416-018-0371-8)
Supplement: Supplementary file 4 — Supplementary table 4 [file 41416_2018_371_MOESM4_ESM.docx]

Supplementary table 4. Hazard ratios per 5 cm increase in height for all cancers and cancers at 23 different sites, stratiﬁed by gender and smoking status.

| Subtype | HR^a^ (95% CI) of men | |  | HR^a^ (95% CI) of women | |
| --- | --- | --- | --- | --- | --- |
|  | Non Smoker | Smoker |  | Non Smoker | Smoker |
| Cancer | 1.06 (1.06, 1.06) | 1.04 (1.04,1.05) |  | 1.11 (1.11,1.11) | 1.11 (1.09,1.13) |
| **Stomach** | 1.02 (1.01,1.03) | 1.00 (0.99,1.01) |  | 1.04 (1.03,1.05) | 1.01 (0.97,1.06) |
| **Colorectum** | 1.05 (1.04, 1.05) | 1.05 (1.04,1.06) |  | 1.10 (1.09,1.11) | 1.10 (1.06,1.14) |
| Liver | 1.01 (1.00,1.02) | 0.98 (0.97,1.00) |  | 1.05 (1.04,1.07) | 1.01 (0.94,1.08) |
| **Pancreas** | 1.04 (1.03,1.06) | 1.03 (1.01,1.05) |  | 1.09 (10.7,1.10) | 1.04 (0.97,1.12) |
| **Lung** | 1.07 (1.06,1.09) | 1.05 (1.03,1.05) |  | 1.12 (1.10,1.13) | 1.08 (1.02,1.14) |
| Breast | . | . |  | 1.17 (1.16,1.17) | 1.17 (1.13,1.21) |
| Cervix uteri | . | . |  | 1.04 (1.03,1.06) | 1.11 (1.04,1.18) |
| Corpus uteri | . |  |  | 1.11(1.09,1.13) | 1.10 (1.00,1.22) |
| **Ovary** | . |  |  | 1.10 (1.08,1.12) | 1.12 (1.03,1.21) |
| Thyroid | 1.19 (1.18,1.21) | 1.21 (1.19,1.23) |  | 1.17 (1.17,1.18) | 1.22 (1.18,1.26) |
| Lymphoma | 1.14 (1.11,1.16) | 1.12 (1.08,1.16) |  | 1.17 (1.15,1.20) | 1.28 (1.12,1.46) |
| **Oral cavity** | 1.03 (1.00,1.06) | 0.99 (0.96,1.02) |  | 1.04 (1.01,1.08) | 1.03 (0.89,1.20) |
| **Esophagus** | 1.01 (0.99,1.04) | 0.98 (0.95,1.01) |  | 1.00 (0.94,1.08) | 0.97 (0.79,1.18) |
| BT/GB | 1.03 (1.01,1.05) | 0.98 (0.96,1.01) |  | 1.03(1.00,1.05) | 0.96 (0.86,1.07) |
| **Larynx** | 1.06 (1.02,1.10) | 1.00 (0.97,1.04) |  | **1.21**(1.08,1.36) | 1.16 (0.89,1.52) |
| **Kidney** | 1.14 (1.12,1.16) | 1.12 (1.10,1.15) |  | 1.16 (1.13,1.19) | 1.21 (1.04,1.41) |
| **Bladder** | 1.09 (1.07,1.11) | 1.06 (1.04, 1.08) |  | 1.08 (1.05,1.11) | 1.08 (0.94,1.25) |
| CNS | 1.06 (1.03,1.09) | 1.08 (1.04,1.12) |  | 1.07 (1.05,1.10) | 1.08 (0.94,1.24) |
| MM | 1.07 (1.03,1.11) | 1.02 (0.97,1.09) |  | 1.10 (1.06,1.14) | 1.07 (0.81,1.42) |
| **Leukemia** | 1.12 (1.09,1.16) | 1.09 (1.05,1.14) |  | 1.09 (1.05,1.12) | 1.17 (0.97,1.40) |
| Skin | 1.08 (1.02,1.14) | 1.06 (0.98,1.15) |  | 1.09 (1.04,1.15) | 0.89 (0.66,1.20) |
| Prostate | 1.06 (1.05, 1.07) | 1.04 (1.03,1.06) |  |  | . |
| Testis | 1.16 (1.10,1.23) | 1.09 (1.01,1.17) |  | . | . |

HR, hazard ratio; CI, confidential interval; CNS, central nervous system; BT, biliary tract; GB, gallbladder; MM, multiple myeloma

^a^Adjusted for age, sex, body mass index, current smoking, current alcohol consumption, regular physical activity and diabetes mellitus

Bold style denotes smoking-related cancers
